# Identification and characterization of extrachromosomal circular DNA in alcohol induced osteonecrosis of femoral head

**DOI:** 10.3389/fgene.2022.918379

**Published:** 2022-09-30

**Authors:** Tingting Zhou, Shiqiang Ma, Yunchao Zhao, Donghui Guo, Hengjun Wang, Mingjie Kuang, Xiaoming Li

**Affiliations:** ^1^ Department of Orthopedics, Traditional Chinese Medicine-Western Medicine Hospital of Cangzhou City, Cangzhou, China; ^2^ Hebei Key Laboratory of Integrated Traditional and Western Medicine in Osteoarthrosis Research (Preparing), Cangzhou, China; ^3^ Department of Orthopedics, Provincial Hospital Affiliated to Shandong First Medical University, Jinan, China

**Keywords:** extrachromosomal circular DNA, alcohol induced osteonecrosis of femoral head, chromosomal instability, bioinformatics, function of eccDNA

## Abstract

Alcohol-induced osteonecrosis of the femoral head (AIONFH) is a complicated refractory bone disease seen in the clinic. The pathogenesis of AIONFH is still controversial. Extrachromosomal circular DNA (eccDNA) elements have been indicated ubiquitously exist in eukaryotic genomes. However, the characteristics and biological functions of eccDNAs remain unclear in AIONFH. In this study, eccDNAs from AIONFH samples (*n* = 7) and fracture of femoral neck samples as a control (*n* = 7) were purified by removing linear DNA and rolling circle amplification. High-throughput sequencing and bioinformatics analysis were performed to study the characterization and biofunction of eccDNAs. We identified more than 600,000 unique eccDNAs. The number of detected eccDNAs in AIONFH was less than that in the control, and eccDNA formation may be related to transcription or other characteristics of coding genes. The eccDNA lengths are mainly distributed between 0.1 kb and 1 kb, with a major peak in 0.358 kb. The bioinformatic analysis showed that 25 significant genes were detected, including *MAP3K1*, *ADCY1*, *CACNA1S*, and *MACF1*, which contributed to regulating bone formation. GO and KEGG analyses suggested that the related genes derived from exons mainly affected metabolic processes and signal transduction, and bone metabolism-related pathways, such as the *MAPK* pathway and *TGF-β* pathway, were enriched. EccDNAs in AIONFH are common and may play an important role in pathogenesis by regulating bone metabolism.

## 1 Introduction

Alcohol-induced osteonecrosis of the femoral head (AIONFH) is a complicated refractory bone disease seen in the clinic, mainly due to long-term and high-dose consumption of alcohol. AIONFH is characterized by bone cell death, which progressed to biomechanical failure and collapse of the femoral head. The symptoms of AIONFH in its early stages are not obvious, and diagnosis of AIONFH occurs late. Collapse of the femoral head is usually observed at the first diagnosis, and patients can be treated only with hip arthroplasty, which increases the economic burden on patients or society. Although the mechanism of AIONFH has been increasingly studied, the pathogenesis of AIONFH is still unclear. Determining the detailed molecular mechanisms or novel stable biomarkers is urgent for the diagnosis and management of AIONFH.

Extrachromosomal circular DNA (eccDNA) elements were first found in 1965 and then were determined to be ubiquitous in eukaryotic genomes, including human genomes (Hotta and Bassel, 1965; Cohen and Segal, 2009). EccDNAs are independent of the chromosome and reflect the plasticity of the genome. Whole-genome sequence analyses of eccDNAs have reported that the sizes of eccDNAs are heterogeneous, ranging from <100 base pairs to megabases (Mb) and carry not only complete or partial genes but also intergenic sequences (Cohen et al., 2008; Cohen et al., 2010; Møller et al., 2015). The biogenesis of eccDNAs has not yet been fully determined. Multiple pathways may correlate with eccDNA formation, such as homologous recombination, microhomology-directed repair, nonhomologous recombination, or R-loop formation (Misra et al., 1989; van Loon et al., 1994; Dillon et al., 2015; Li et al., 2022).

An increasing number of eccDNA studies have proven that eccDNAs contribute to tumorigenesis and drug resistance. In cancer, more than 100 kb of extrachromosomal DNA (called ecDNA, such as double minute chromosomes) amplification increases oncogene copy numbers and intratumoral heterogeneity (Turner et al., 2017; Verhaak et al., 2019; Wu et al., 2019). The dynamic regulation of oncogenic variant EGFR-vIII from ecDNA is correlated with resistance to targeted therapies (Nathanson et al., 2014). ERBB2/EGFR were present as eccDNA in gastric cardia adenocarcinoma and formed frequent local amplifications, which was associated with prognosis (Zhao et al., 2021) In contrast to larger eccDNAs (>100 kb, ecDNAs), smaller eccDNAs (<100 kb) are common in human cells, including normal cells. The eccDNAs in circulation that are released by normal or cancerous tissue contribute to the diagnosis of disease or intercellular communication (Kumar et al., 2017). Furthermore, eccDNAs are also common in human somatic tissues, and they may affect phenotypes by regulating genes (Møller et al., 2018). And the small eccDNA in mammalian tissues and cell lines may modulate gene expression through the production of both known and novel regulatory small RNA (Paulsen et al., 2019). Although eccDNAs have been detected in cancer, plasma, muscle, and leukocytes, characterization of eccDNA in the human femoral head has not been reported. In this study, we utilized the circle-seq method to characterize and identify the biological function of eccDNA in the femoral head (femoral neck fracture or osteonecrosis of the femoral head).

## 2 Materials and methods

### 2.1 Sample collection and preparation

Femoral head samples of femoral neck fractures and AIONFH were collected through total hip arthroplasty at the Cangzhou Hospital of Integrated TCM-WM Hebei. All procedures were approved by the ethics committee of the Cangzhou Hospital of Integrated TCM-WM Hebei (NO. 2018033) and complied with the World Medical Association Declaration of Helsinki. The control group included seven fracture patients, aged 71.86 ± 8.10 years and weighing 64.71 ± 8.97 years, and the AIONFH group included seven AIONFH patients, aged 52.86 ± 12.32 years and weighing 68.57 ± 7.89 years. The bone samples in the control group and necrotic bone in the AIONFH group were harvested, quickly transferred into liquid nitrogen, and later stored at -80°C. The screening workflow was showed in supplement Figure 1.

**FIGURE 1:**
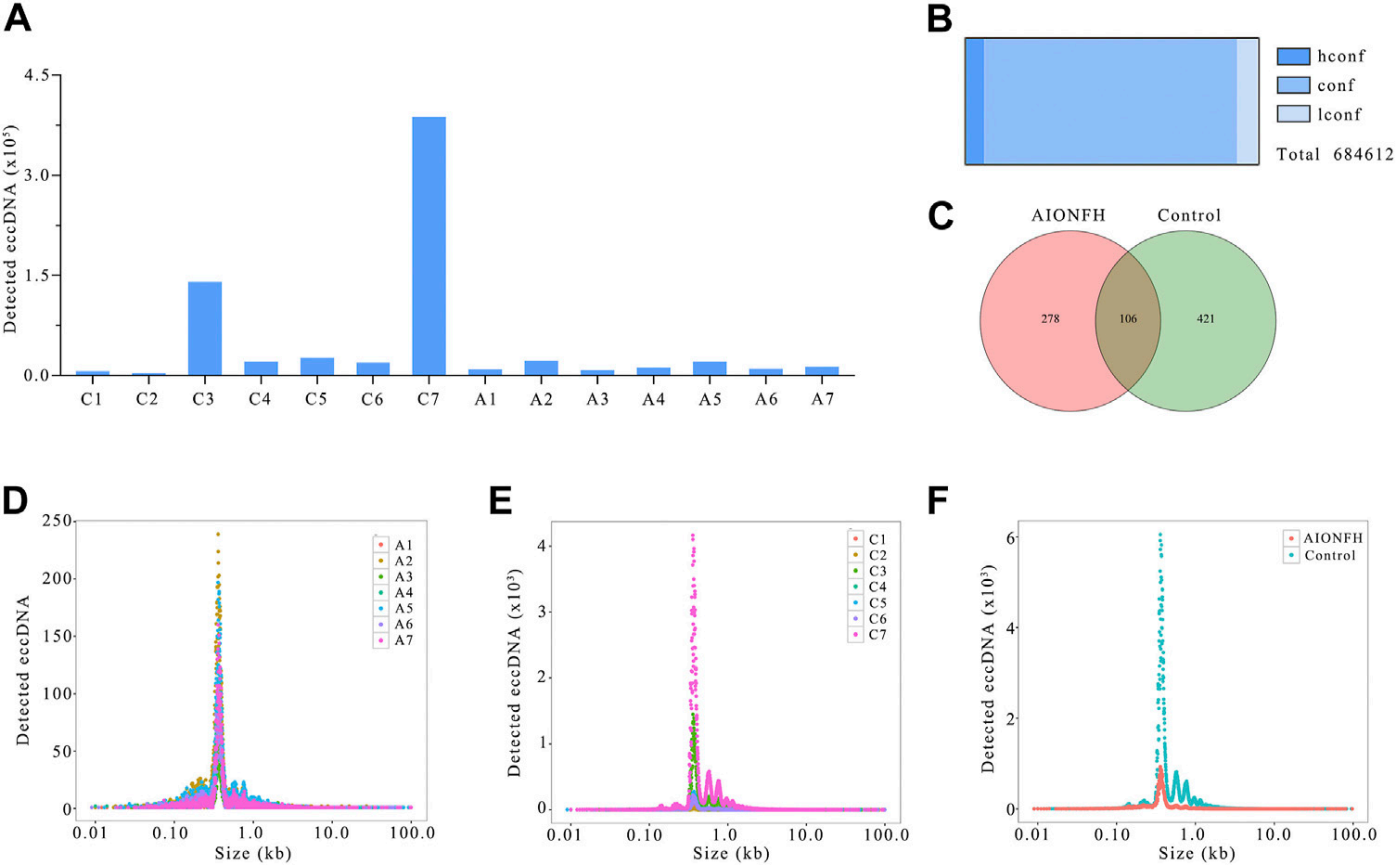
EccDNAs number, sizes distribution of AIONFH group and control group. **(A)**. The total amount of eccDNAs detected in each sample. **(B)**. Confidence of identified eccDNAs. **(C)**. Venn diagram showed the consist eccDNAs detected in AIONFH and control samples. **(D–F)**. The length distribution of eccDNAs (<100 kb) in each group.

### 2.2 Extrachromosomal circle DNA enrichment

5-mg tissue samples were ground with a freezing grinder and then transferred to 1.5-ml centrifuge tubes. DNA was extracted following the protocol of the HiPure Tissue and Blood DNA Kit (Magen #D3018). 200 μl of Buffer ATL and 20 μl of Proteinase K were added to each sample, which was followed by incubation with shaking at 55°C for 60 min. RNase A (10 μl) was used to remove RNA. 200 μl of Buffer AL was added to each sample, which was followed by high-speed eddying for 10 s and incubation with shaking at 65°C for 10 min. Then, absolute ethanol and high-speed eddying were added for 10 s, transferred to the Hipure DNA Mini Column I, and centrifuged at 10000 × g for 1 min. Then, 500 μl of washing buffer GW1 (diluted with absolute ethanol) was added to the column and centrifuged at 10,000 × g for 1 min, and 650 μl of washing buffer GW2 was added to the column and centrifuged at 10,000 × g for 1 min. The effluent was discarded, the column was reinstalled into a new collection tube, and centrifuging was conducted at 10,000 × g for 3 min. The column was placed in a new centrifuge tube, 50 μl of buffer AE was added to the center of the column membrane for 3 min, and the sample was then centrifuged at 10,000 × g for 1 min. The DNA samples were stored at 4°C and for long-term storage at -20°C.

### 2.3 Removal of linear and mitochondrial DNA

Linear DNA was removed by exonuclease. The detailed protocol consisted of taking 1 μg of DNA and adding 2 μL of Plasmid Safe ATP-dependent DNase (Epicenter) and incubating this mixture at 37°C for 16 h. Fifty microliters of DNA Clean Beads were added to the reaction product obtained from the previous step for purification and transferred to a magnetic stand after 2 min at room temperature. Freshly prepared 80% ethanol was used to rinse the magnetic beads twice. Finally, ddH2O was added to elute DNA after removing the supernatant.

### 2.4 Circle-sequence

The DNA samples were used with a TruePrep DNA Library Prep Kit V2 for Illumina (Vazyme #TD501) to build the library and VAHTS DNA Clean Beads (Vazyme #N411) for purification. The DNA libraries of 14 samples were sequenced with high-throughput sequencing technology, and each sample contained 30 G of data. The original data obtained by sequencing were converted into sequence data by base calling, which we refer to as raw data or raw reads, and the results were stored in FASTQ (Chen et al., 2018) format after filtering of low-quality data.

### 2.5 Split junction sequence verification

EccDNAs were detected by Circle-map (Møller et al., 2018) software. The basic principle of Circle-map is to use the discordant read pair to initially locate the position of the circular DNA interface and then use a soft clipped read (split read) to determine the exact position of the circular DNA interface. Each detected circular DNA structure was supported by a minimum of two independent structural-read variants, such as one split read and one discordant read pair. Both discordant read pairs and split reads were used for the quantification of circular DNA. In addition, the software also considers factors such as the coverage of sequencing reads in the circular DNA interval and changes in the sequencing depth compared to the surrounding interval to determine the confidence of the circular DNA detection. We rank the credibility of eccDNA based on the above-mentioned indicators: hconf = high confidence, conf = confidence, and lconf = low confidence; 1) low confidence eccDNA is found in at least 1 sample and meets one of the following conditions: a) Supported split reads ≥ 1; 2) Confident eccDNA is found in at least 1 sample and the following two conditions are met: a) supported split reads≥1; b) the sequencing coverage of the corresponding eccDNA region is ≥ 80%; 3) high-confidence eccDNA meets the following three conditions in at least one sample a) supported split reads≥1; b) the sequencing coverage of the corresponding eccDNA region is ≥ 80%; c) the relative sequencing depth (copy number) of the eccDNA region is more than twice the average level of other regions of the genome (i.e., the coverage at the start and coverage at the end is greater than 0.5).

### 2.6 Validation of eccDNA through polymerase chain reaction and Sanger sequencing

Experimental validation was performed on four significantly different expression eccDNAs of clinic samples. DNA was extracted from the clinic specimens and the eccDNA extract method was the same with described above. PCR was performed using Accurate Taq Master Mix (dye plus) (Accurate Biotechnology, China) to assess the expression levels. The reaction conditions were 94°C for 30 s, 28 cycles at 98°C for 10 s, 55°C for 30 s, and 72°C for 1 min, followed by final elongation at 72°C for 2 min and storage at 4°C. The primers of the eccDNAs were designed using the “outward” directing strategy and described in Supplementary Table S1. PCR products were loaded onto 1.5% agarose gels and visualized under an ultraviolet Luminescent Image Analyzer (GE Healthcare Life Sciences, United States). The PCR product were sent for Sanger sequencing.

### 2.7 Enrichment analysis and functional annotation of eccDNA

To further estimate the biofunction and signaling pathways as well as the eccDNAs, we performed bioinformatics analyses. EdgeR software were used to calculate the *p* values of different eccDNA the false discovery rate (FDR) was used to correct the *p* values. The different eccDNAs were identified using *p* < 0.05 and |log2FC|>1. The gene ontology enrichment analysis and KEGG pathway enrichment analysis were based on the NCBI database.

## 3 Results

### 3.1 The amount and length distribution of extrachromosomal circular DNA

To identify the features of eccDNAs in the human femoral head, we completed eccDNA high-throughput sequencing by using circle-seq on a genomic scale. EccDNA was detected in 14 femoral head samples (mean age 62.36), including 7 femoral neck fracture samples (mean age 71.86), which represented normal tissue as the control group, and in 7 AIONFH samples (mean age 52.86) as the AIONFH group. The purification, enrichment, and identification of eccDNAs from the femoral head are described in the Materials and Methods section. Based on previous studies, we first analyzed eccDNAs at a cutoff point of 100 kb. EccDNAs with lengths greater than 100 kb were called ecDNAs in this study. We also assessed the confidence of detecting eccDNAs.

In total, 684612 eccDNAs annotated to 23 pairs of chromosomes were detected in 14 samples. The number of eccDNAs in the control group (*n* = 596621) was greater than that in the AIONFH group (*n* = 88720), and individual variations were present within the two groups (Figure 1A). The eccDNAs classes included 43088 hconf, 591417 conf, and only 50107 lconf (Figure 1B). Approximately 93% belonged to the conf or hconf classes. Consistent eccDNA was detected in at least two samples in one group, and the common and unique eccDNAs between the AIONFH and control groups were then compared. The Venn diagram showed that out of 805 eccDNAs, 278 eccDNAs were detected only in the AIONFH group, 421 were detected only in the control group, and 106 eccDNAs were detected in both groups (Figure 1C). These specific eccDNAs may be potential biomarkers for the early diagnosis of AIONFH. The length distributions in the AIONFH and control groups showed similar features. The overall length distribution of eccDNAs ranged in size from 0.9 kb to 99.7 kb, with a distinctive peak at 0.359 kb in the AIONFH group. Interestingly, there was a similar major peak at 0.358 kb in the control group compared with the AIONFH group (Figure 1D–F). In total, the vast majority of eccDNAs (85.7%) were smaller than 3 kb.

**FIGURE 2:**
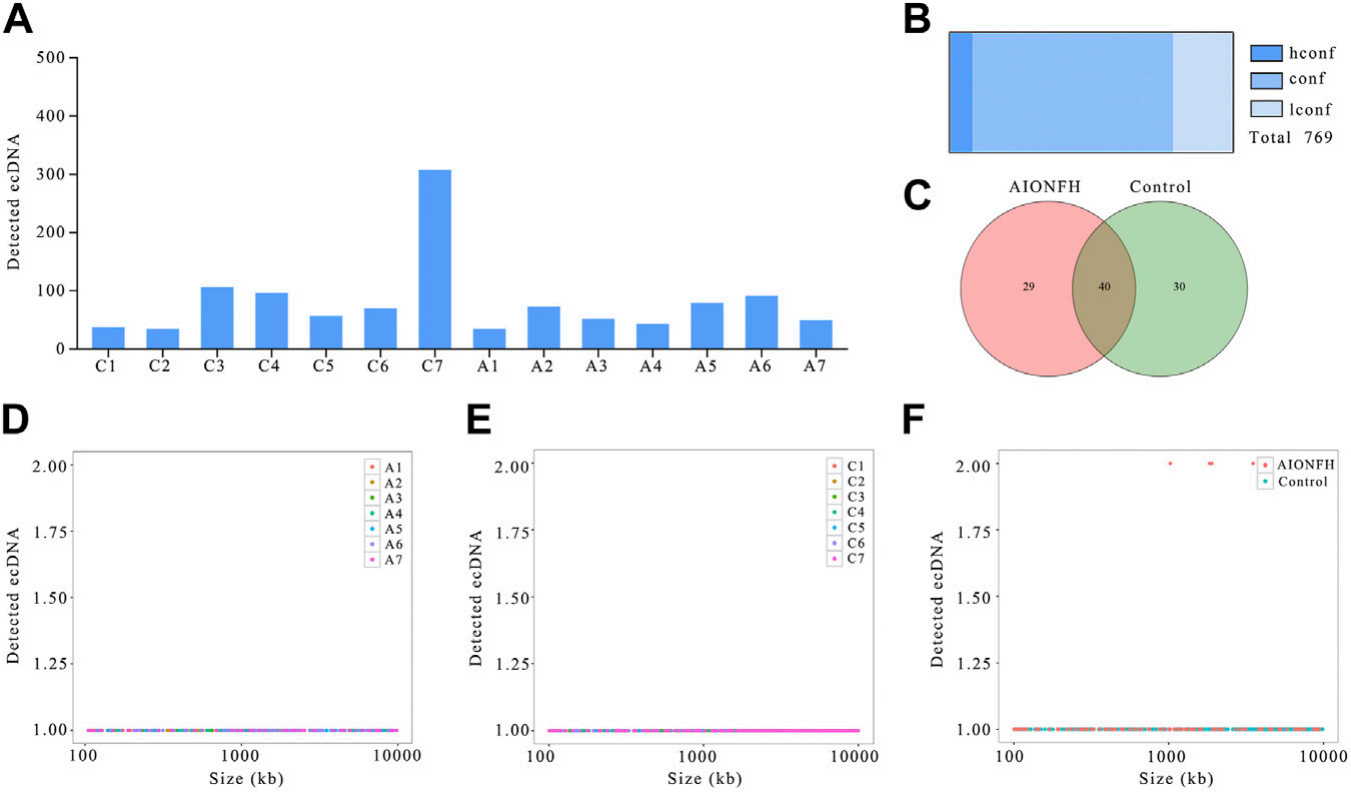
EcDNAs number, sizes distribution of AIONFH group and control group. **(A)**. The total amount of ecDNAs detected in each sample. **(B)**. Confidence of identified ecDNAs. **(C)**. Venn diagram showed the consist ecDNAs detected in AIONFH and control samples. **(D–F)**. The length distribution of ecDNAs (100kb–10000 kb) in each group.

We detected 769 ecDNAs that have eccDNA lengths greater than 100 kb in 14 samples, which means that ecDNAs exist not only in tumor tissue but also in the human femoral head. However, the number of ecDNAs was significantly less than that of eccDNAs, which was also consistent with the results of previous studies. There were more EcDNAs in the control group (*n* = 587) than in the AIONFH group (*n* = 294), which was similar to the results for eccDNAs, but the differences between the control group and AIONFH group were less than those of the eccDNAs (Figure 2A). The majority of ecDNAs were conf (*n* = 545) or hconf (*n* = 62). Only 162 lconf ecDNAs were detected (Figure 2B). The Venn diagram showed that out of 99 ecDNAs, 29 ecDNAs were detected only in the AIONFH group, 30 were detected only in the control group, and 40 eccDNAs were detected in both groups (Figure 2C). The ecDNA lengths in the two groups ranged from 100.5 kb to 998.83 kb, and the frequencies of the lengths were well distributed without any peaks, which was different from eccDNAs (Figure 2D–F).

We have screened the differential expressed eccDNA and confirmed four significantly expressed eccDNAs in clinic samples using outward PCR and Sanger sequencing (supplement Figure 3) and named E1(chr2:16225125–16226720), E2(chr10:125824765–125825185), E3(chr23440318-23440960), E4(chr2:88832773–88859607).

**FIGURE 3:**
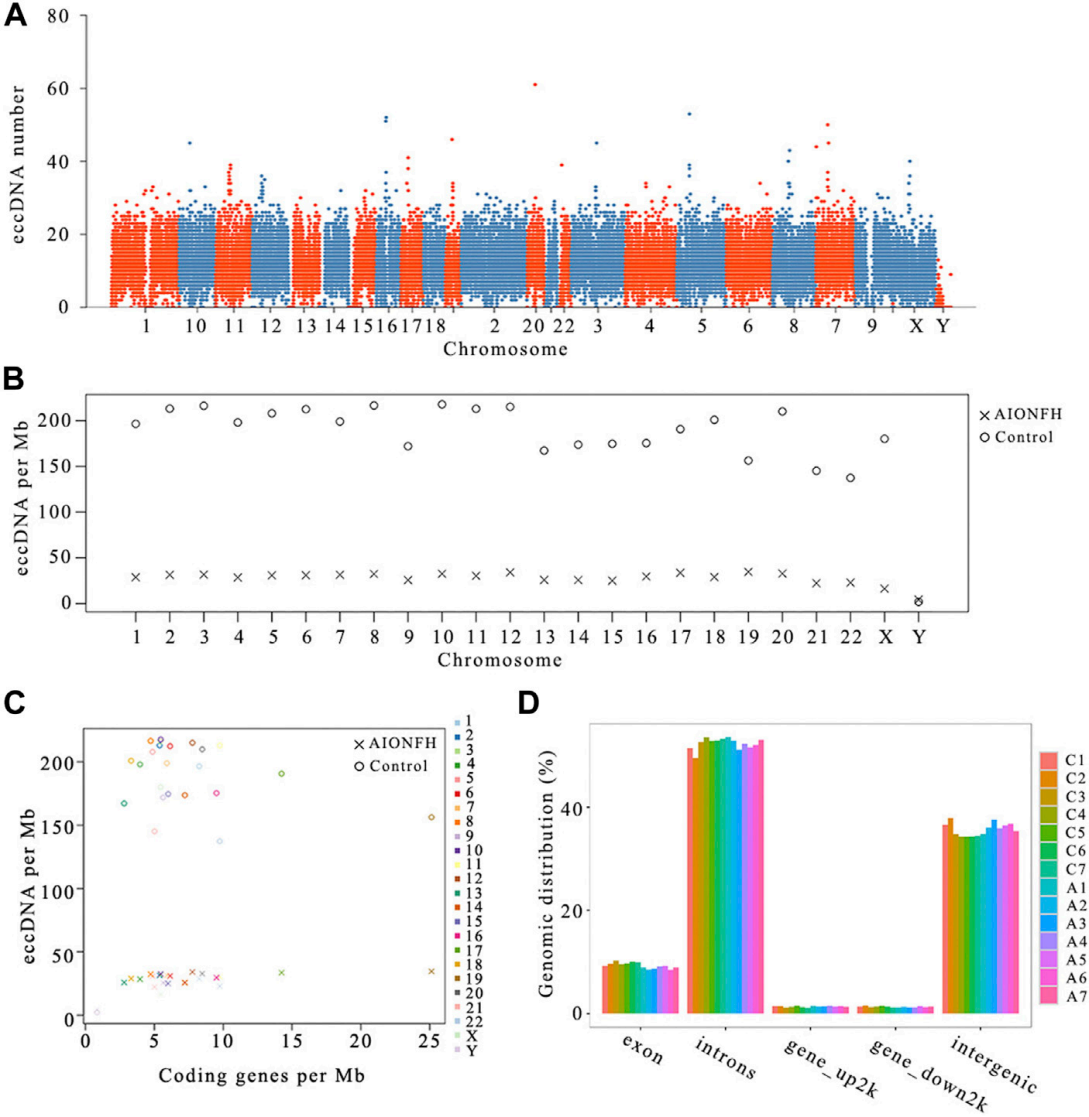
Genomic distribution features of eccDNAs between AIONFH and control. **(A–B)**. The distribution of eccDNAs on chromosomes. **(C)**. The origin region distribution of eccDNA on the genome. **(D)**. the correlation of eccDNA per Mb with coding genes per Mb.

### 3.2 Genomic distribution pattern of eccDNAs between alcohol-induced osteonecrosis of the femoral head group and control group

We detected the amounts of eccDNAs in whole genomes with a range of 50 kb and mapped the overall population of femoral head eccDNAs annotated to 23 paired chromosomes. The distribution pattern of the eccDNAs revealed that they were common in each of the 23 pairs of chromosomes. Chromosomes 1 and 2 formed more eccDNAs (Figure 3A). The eccDNA frequency per Mb in the control group was higher than that in the AIONFH group. The amounts of eccDNA per Mb in chromosomes 3, 8, and 10 were higher than those of the other chromosomes in the control group. The eccDNA frequency per Mb was comparable in the AIONFH group except for chromosome Y. Chromosome Y had a much lower frequency of eccDNAs in both groups. Chromosomes with higher eccDNA/Mb in the AIONFH group were the gene-rich chromosomes 19, 12, and 17. We explored the correlation of the eccDNA/Mb ratio and coding genes/Mb in the AIONFH group and control group. Interestingly, we found that there was a significantly positive correlation of eccDNA/Mb with coding genes/Mb in the AIONFH group (*p* = 0.033) and no significant correlation in the control group (*p* = 0.681). The gene-rich chromosomes 19 and 17 in the AIONFH group coded more genes and generated more eccDNAs, which suggested that transcription or other characteristics of coding genes may contribute to the formation of eccDNAs in the AIONFH group (Figures 3B,C). The eccDNAs in the femoral head were enriched in exons, introns, gene_up2k, gene_down2k, and intergenic regions, and the source of gene distribution was not uniform. The eccDNAs mainly originated from introns, intergenic regions, or exon regions. However, eccDNAs rarely formed from gene_up2k or gene_down2k (Figure 3D).

### 3.3 Genomic distribution of ecDNAs and the correlation with frequency of ecDNAs

**FIGURE 4:**
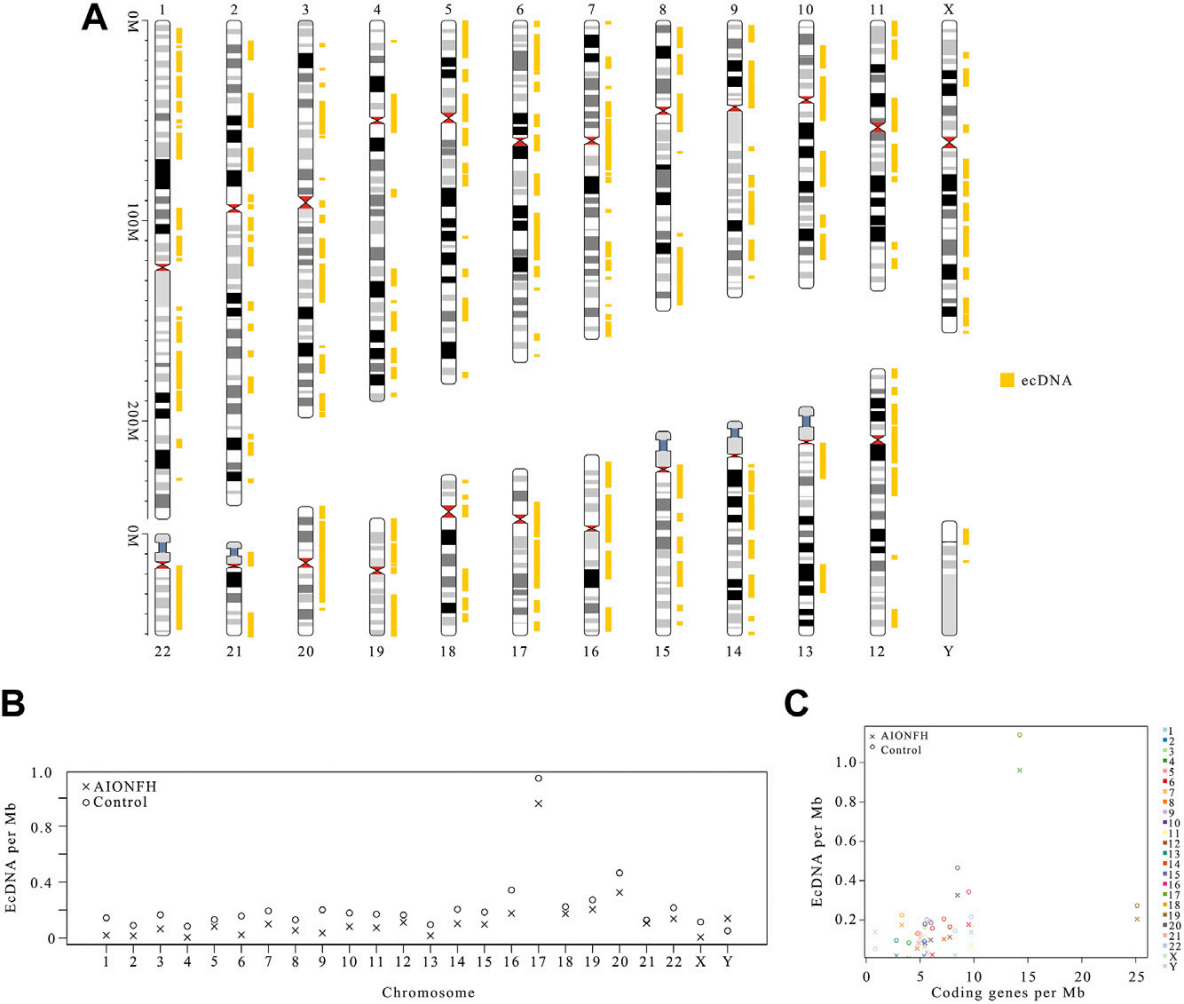
Genomic distribution of ecDNAs and the correlation with frequency of ecDNAs. **(A)**. The distribution of ecDNA fragment in chromosomes. EcDNA per Mb of AIONFH group and control group: chromosomes **(B)** and coding genes per Mb **(C)**.

We mapped the distribution of ecDNA (>100 kb) in chromosomes and analyzed the ecDNA per Mb in chromosomes (Figure 4A). The genetic distribution of ecDNAs was mainly concentrated in exons and intergenic regions, which was different from the eccDNAs. The highest density of ecDNA per Mb was observed for chromosome 17, which was 5.2-fold higher than the average level of the other chromosomes in the control group and 7.38-fold higher than the average level of the other chromosomes in the AIONFH group (Figure 4B). The densities of ecDNAs in chromosomes 19 and 20 were also higher than those in the other chromosomes. There was a significant weak correlation of ecDNA per Mb with coding genes per Mb in both the control group (r = 0.480, *p* = 0.018) and AIONFH group (r = 0.444, *p* = 0.030), which means that transcription or other characteristics of coding genes may affect the formation of ecDNAs, similar to eccDNA (*p* < 0.05) (Figure 4C).

### 3.4 Bioinformatic analysis of functional attributes connected to eccDNAs changes in gene sets and signaling pathways

**FIGURE 5:**
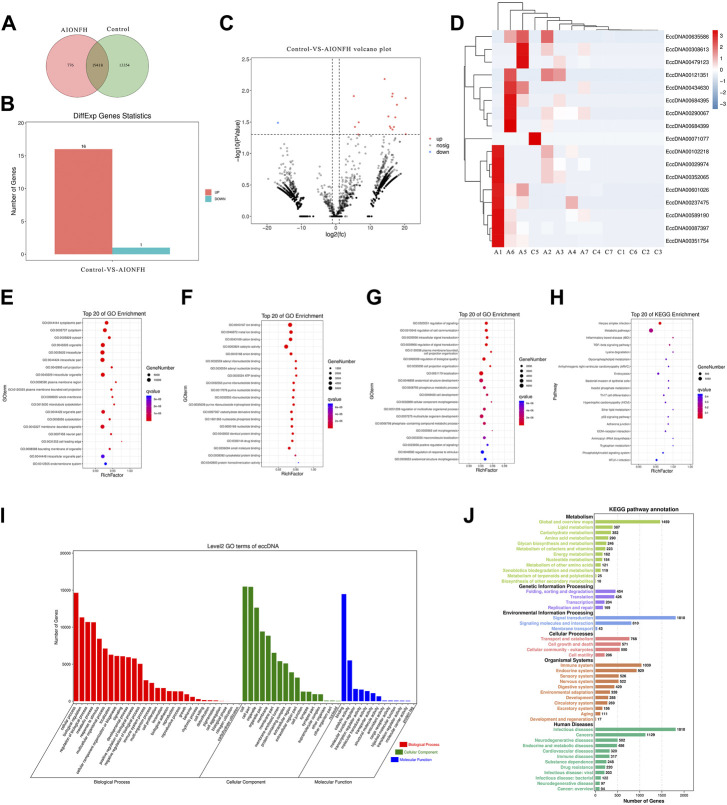
Bioinformatic analysis of functional attributes connected to eccDNAs changes in gene sets and signaling pathways. **(A)**. Common and specific characteristics of eccDNAs of AIONFH group and control group were showed in Venn diagram. **(B)**. Upregulated and downregulated eccDNAs related genes. **(C,D)**. Volcano plot and hot map show the different genes. **(E,F)**. Top 20 of Gene ontology terms (Biological Processed, Molecular Functions, Cellular Components) enriched in the plot figure. **(I)**. GO biological process classification of genes. **(H)**. Top 20 of KEGG enrichment signal pathways. **(J)**. KEGG function enrichment analysis.

We explored the biological functions of the eccDNA-related encoding genes because most extrachromosomal circular DNA elements were less than 100 kb in length in the human femoral head. Common or specific analyses of the eccDNA-related genes in the two groups are displayed in a Venn diagram, which reveals that 19418 eccDNA-related genes were identical in the two groups. The differentially expressed eccDNA-related genes in the AIONFH group and control group were also investigated, and the results showed that 776 eccDNA-related genes were specific to the AIONFH group, 13354 eccDNA-related genes were unique to the control group, and 19458 eccDNA-related genes were detected in both the AIONFH and control groups (Figure 5A). The differential gene expressions in eccDNAs were illustrated *via* a histogram, hot map, and volcano plot. Sixteen genes were upregulated, and only 1 gene was downregulated (Figures 5B–D). The cellular component categories, molecular functions and biological processes revealed that eccDNA-related gene networks play a key role in signal transduction and metabolic processes (Figures 5E–H). KEGG pathway enrichment analyses were performed to determine the functional features, and the results indicated that related genes were more abundant in the signal transduction, infectious diseases, and metabolic pathways. The TGF-β signaling pathway was enriched by eccDNA-related genes, which contributed to osteogenic differentiation.

### 3.5 Bioinformatic analysis of exon region of gene in eccDNAs

**FIGURE 6:**
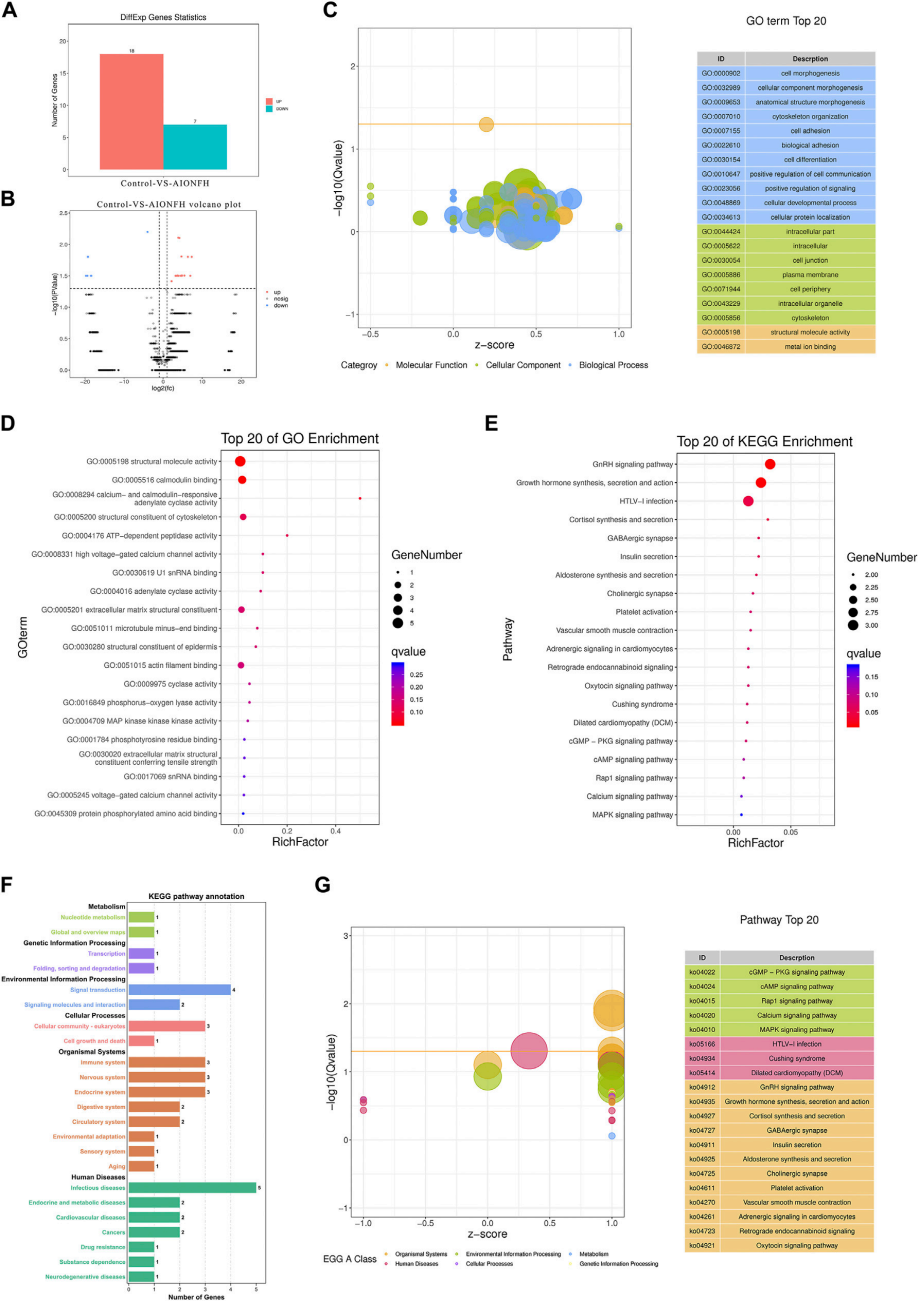
Bioinformatic analysis of exon region of gene in eccDNAs. **(A)**. Upregulated and downregulated eccDNAs related exon region genes. **(B)**. Volcano plot shows the different genes. **(C,D)**. GO biological process classification of genes and top 20 of GO terms enriched in the plot Figure **(E–G)**. KEGG function enrichment analysis.

We explored the differences in eccDNA-related genes that were mainly derived from exons and evaluated their biological functions to exclude the interference of introns and intergenic regions. As shown in Table 1, 25 significant genes were detected. A total of 18 targets were upregulated, including *LORICRIN, CNTNAP2, ZNF431, MAP3K1, ADCY1,* and CACNA1S, and 7 targets were downregulated, such as *MACF1,* in the AIONFH group compared to the control group (Figures 6A,B). Previous studies have proven that *MAP3K1, CACNA1S, COL12A1,* and *MACF1* contribute to bone formation, which indicates that eccDNA-containing exon-region genes may regulate osteogenesis in AIONFH. Gene ontology enrichment analyses suggested that related genes derived from exons mainly affected metabolic processes and signal transduction (Figures 6C,D). We also conducted KEGG pathway enrichment analysis to evaluate the characteristics of the eccDNA(exon)-related gene expressions, and the results revealed that the *MAPK* pathway, *GnRH* signaling pathway, oxytocin signaling pathway, and *cGMP-PKG* signaling pathway were enriched. Related genes were more abundant in infectious diseases and environmental information processing (Figures 6E–G).

**TABLE 1:** Significant exon region genes of eccDNAs.

Id	Control	AIONFH	log2(fc)	PValue	FDR	Symbol
ENSG00000127603	896.1243	56.55429	−3.98599	0.006348	1	*MACF1*
ENSG00000203782	41.74714	616.2157	3.883686	0.007813	1	*LORICRIN*
ENSG00000174469	34.36	610.1686	4.150406	0.007913	1	*CNTNAP2*
ENSG00000196705	20.50571	535.6743	4.707258	0.015625	1	*ZNF431*
ENSG00000127481	642.4114	0.001	−19.2931	0.015776	1	*UBR4*
ENSG00000171914	615.4014	0.001	−19.2312	0.015776	1	*TLN2*
ENSG00000197245	2.771429	445.0943	7.327337	0.015776	1	*FAM110D*
ENSG00000282872	5.542857	445.0943	6.327337	0.015776	1	*C1orf232*
ENSG00000095015	18.56571	481.4043	4.696536	0.03125	1	*MAP3K1*
ENSG00000164742	15.79429	457.4171	4.856036	0.03125	1	*ADCY1*
ENSG00000176771	29.65	371.8471	3.648606	0.03125	1	*NCKAP5*
ENSG00000229017	11.08429	481.4043	5.440661	0.03125	1	*-*
ENSG00000081248	34.36	470.8943	3.776601	0.031466	1	*CACNA1S*
ENSG00000111799	28.81857	407.4486	3.821547	0.031466	1	*COL12A1*
ENSG00000137936	369.0429	0.001	−18.4934	0.031466	1	*BCAR3*
ENSG00000146416	21.33714	488.91	4.51813	0.031466	1	*AIG1*
ENSG00000152591	17.73429	485.6657	4.775351	0.031466	1	*DSPP*
ENSG00000175718	44.51857	468.0629	3.394223	0.031466	1	*RBMXL3*
ENSG00000177694	699.3343	0.001	−19.4156	0.031466	1	*NAALADL2*
ENSG00000182095	2.771429	342.3229	6.948584	0.031466	1	*TNRC18*
ENSG00000225937	343.4171	0.001	−18.3896	0.031466	1	*-*
ENSG00000234170	885.8443	0.001	−19.7567	0.031466	1	*-*
ENSG00000242086	49.22857	438.5086	3.155037	0.031466	1	*-*
ENSG00000249001	30.75857	485.6657	3.980903	0.031466	1	*-*
ENSG00000102910	181.8057	804.5586	2.1458	0.038574	1	*LONP2*

### 3.6 Correlation analysis of eccDNAs and ecDNA quantity between alcohol-induced osteonecrosis of the femoral head group and control group

**FIGURE 7:**
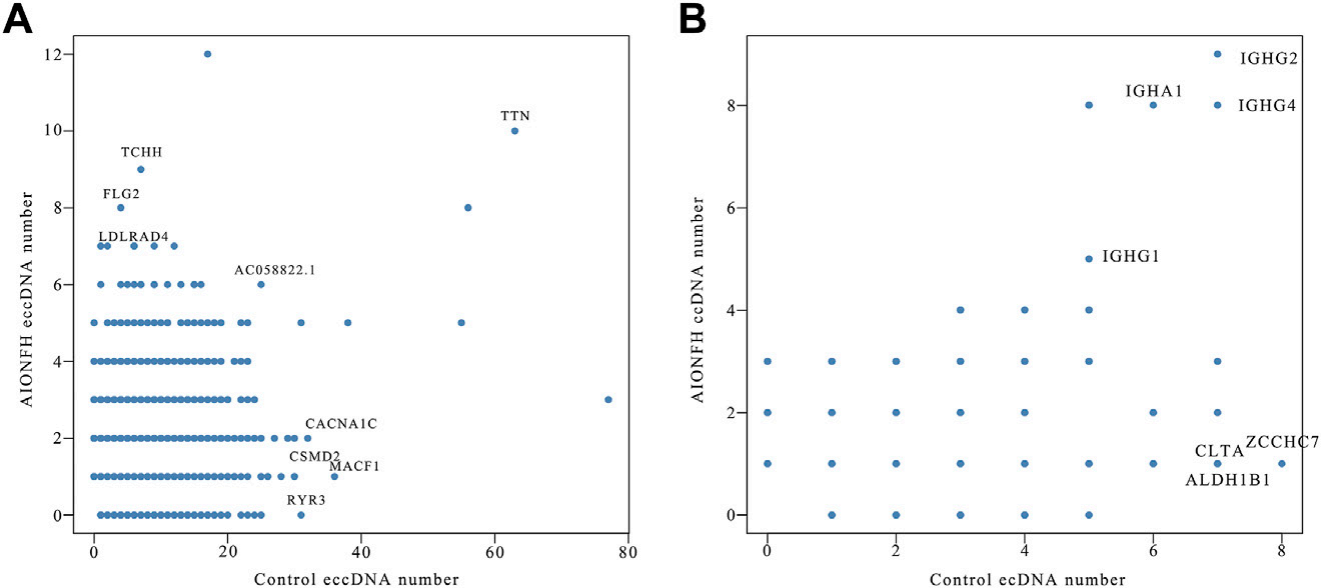
Correlation analysis of eccDNAs **(A)** and ecDNA **(B)** quantity between AIONFH group and control group.

To explore the correlation between the AIONFH group and control group in terms of the amounts of eccDNAs per gene, we calculated the amounts of eccDNAs in each gene. The results are illustrated in a scatter plot. We observed a positive correlation between the AIONFH group and control group in terms of the amount of eccDNA per gene; however, this correlation was weak (r = 0.317, *p* < 0.001). *TTN,* which encodes titin, produced mostly eccDNAs in both the AIONFH group and control group. Furthermore, *MACF1* produced more eccDNAs in the control group but fewer in the AIONFH group, which may be the reason for the abnormal osteogenesis in AIONFH (Figure 7A). Similarly, there was no correlation between the AIONFH group and control group in the amount of ecDNA per gene (r = 0.118, *p* < 0.05). The amount of ecDNA derived from *IGHG* was higher in the AIONFH group and control group (Figure 7B).

## 4 Discussion

In the past few years, extrachromosomal circular DNA elements have been widely observed in nearly all eukaryotic cells, including those of humans. Biological function studies of extrachromosomal circle DNA elements have usually focused on cancer, such as contributions to the amplification of oncogenes (Morton et al., 2019), potential biomarkers (Paulsen et al., 2018; Lv et al., 2022), and tumor resistance (Nathanson et al., 2014; Lin et al., 2022). In our study, we reported the features of extrachromosomal circle DNA, distributions, and biological functions in osteonecrosis of the femoral head compared with fractures by using circle-seq, which provided a novel eccDNA map for human disease except cancer. The identification of extrachromosomal circular DNA provides a novel perspective on the mechanism of AIONFH.

Humans can live with Mb-sized circular chromosomes in somatic cells (Møller et al., 2018; Paulsen et al., 2018). In human tumor cells, extrachromosomal circular DNAs usually range from 1 Mb to 3 Mb in size, contain proto-oncogenes and are visible when using light microscopy (Storlazzi et al., 2010; Sanborn et al., 2013; Verhaak et al., 2019). In addition, identification of eccDNAs ranging in size from 0.1 kb to 2 kb is now possible due to advances in sequencing technologies. Most eccDNAs were less than 1 kb (usually 200–400 bp in size) and were visible by using electron microscopy (Shibata et al., 2012). In our study, we identified 685381 unique extrachromosomal circular DNA elements in 14 samples in the two groups, with a major peak at 358 bp. In agreement with previous studies, we found that extrachromosomal circular DNA elements smaller than 100 kb (eccDNA, *n* = 684612) were much more abundant than DNA larger than 100 kb (ecDNA, *n* = 769), mostly 300–400 bp in size. There were no obvious differences in the eccDNA length distributions between the AIONFH group and control group, which was different from that of tumor cells.

We detected more than hundreds of thousands of eccDNAs, which was much larger than the number of eccDNAs (detected in at least two samples in one group). Most eccDNAs were present in either the AIONFH group or control group; only 278 eccDNAs were present in the AIONFH group, and 421 eccDNAs were present in the control group. Although these consistent eccDNAs may provide a possibility for early diagnosis, biomarkers for early AIONFH diagnosis still need to be further tested due to the individual variations in eccDNAs in the two groups. Therefore, we re-screened the detected eccDNAs, and there were 309 eccDNA detected in at least three samples. The different expression of genes in eccDNAs was shown as supplement Figure 4. 12 genes were significantly up-regulated and 10 genes were down-regulate. GO analysis were revealed that eccDNA-related genes were enriched in cellular process, biological regulation and metabolic process. KEGG pathway enrichment analyses indicated that related genes were more abundant in porphyrin and chlorophyll metabolism, transcriptional regulation and metabolic pathways.

The mechanism of eccDNA formation is still unclear. Direct repeat sequences (Cohen et al., 2010), nonhomologous end-joining (Shibata et al., 2012; Zhu et al., 2017), nonallelic homologous recombination (Wang et al., 2004), or microhomology-mediated end-joining (Paulsen et al., 2021) were reported to generate eccDNAs. The generation of eccDNA in germline inversely correlates with the meiotic recombination rate, and chromosomes with high coding-gene density and Alu element abundance form the least eccDNA (Henriksen et al., 2022). Møller HD et al. reported that eccDNAs were mainly mapped to gene-rich chromosomes in healthy human somatic tissues (Møller et al., 2018); however, they were not obvious in tumors. Sun et al. showed that eccDNAs could be mapped to 23 pairs of chromosomes in esophageal squamous cell carcinoma, and eccDNA formation was not connected with chromosomes or coding genes. In our study, higher frequencies of eccDNA per gene were also mapped to gene-rich chromosomes 17 and 19, which means that the eccDNA features were similar to those in nononcological cells. The eccDNA chromosome density of the control was higher than that of AIONFH, except for chromosome Y. Chromosome Y had a much lower frequency of eccDNA in both groups, perhaps due to fewer genes or the denser structure of chromosome Y (Sun et al., 2021). The smaller-sized eccDNAs were mostly derived from sequences in genic regions. The eccDNAs of plasma in pregnant women were generated from 5′-untranslated regions (5′UTR), exon regions, and intron regions (Sin et al., 2020). In our study, eccDNAs were generated from exons, introns, and intergenic regions and rarely originated from geneup_2 k or genedown_2 k, and there was a significantly positive correlation between eccsDNA per Mb with coding genes per Mb, which means that the frequency of eccDNA formation may be associated with chromosomes or coding genes. The *TTN* gene contains a long sequence, which codes titin, which is the most abundant protein in the human body (Savarese et al., 2018). Interestingly, we found that the number of eccDNAs derived from the *TTN* gene was large in both the AIONFH group and control group, which further supports that the transcription, mutation, or deletion of genes may contribute to eccDNA formation.

Most studies have linked the biological functions of eccDNAs with the cancer field, such as intercellular genetic heterogeneity, oncogene amplification, drug resistance, cellular senescence of tumor cells and proinflammatory response (Liao et al., 2020; Li et al., 2022). Little is known about their functions in other diseases. Hence, we explored the different target genes and relevant biological functions of eccDNAs *via* bioinformatic analyses. EccDNA expressions varied between the AIONFH group and control group. A total of 25 eccDNA-relevant target genes were detected, including *MACF1* (Hu et al., 2017), *MAP3K1* (Yang et al., 2018), *CACNA1S* (Kim et al., 2015), and *COL12A1* (Izu et al., 2016), which were correlated with osteogenesis. GO enrichment and KEGG analyses indicated that relevant genes involved in endocrine and metabolic diseases participated in signaling pathways such as the *TGF-β* signaling pathway (Chen et al., 2012) and *MAPK* pathway (Yang et al., 2018), which are also related to bone formation. Alcohol is known to inhibit the activity of osteoblasts and osteogenic differentiation of bone marrow mesenchymal stem cells in AIONFH (Klein et al., 1996; Yang et al., 2019). EccDNAs in AIONFH mainly originate from exons, introns, or intergenic regions, and it is possible that they regulate osteogenesis-related genes such as transcripts or miRNAs. We inferred that eccDNAs may contribute to regulating bone metabolism and participate in the development and progression of AIONFH through the deletion or amplification of eccDNA.

Long-read sequencing technologies is contributed to detect the accurate completly eccDNA sequence. At present, our library construction method of identified eccDNA is based on the reads covered by the circular DNA splicing position. Long-read sequencing will play a key role to further study the biofunction of eccDNA. Complete sequence information of eccDNA will be obtained by long-read sequencing, and structural variation can be observed to analyze the differential functions of eccDNA (Garg, 2021). In this study, the landmark eccDNAs were screened to provide eccDNA database for subsequent diagnosis and treatment of diseases, further linking the function of structural variation to disease requires long-length sequencing to accurately uncover its biofunctions of the sequence.

In summary, we applied a novel sequencing technology to identify and characterize eccDNA features in alcohol-induced osteonecrosis of the femoral head for the first time. We identified hundreds of thousands of eccDNAs with a broad range of sizes and a major peak at 358 bp. We also found that the transcription or other characteristics of coding genes may contribute to the formation of eccDNAs by analyzing the distribution of eccDNAs. In addition, the bioinformatic analyses indicated that the different eccDNA genes in the AIONFH group and control group were involved in some biological functions, such as regulating bone metabolism. The detailed molecular mechanism of eccDNAs in AIONFH merits further exploration.

## Data Availability

Sequence data from Circle-Seq have been deposited in the Genome Sequence Archive, the accession number: HRA003059.
