# Supplementary material for: Identification and characterization of extrachromosomal circular DNA in alcohol induced osteonecrosis of femoral head
Source: Front Genet. 2022 Sep 30;13:918379. doi: 10.3389/fgene.2022.918379 (PMC9561878; doi:10.3389/fgene.2022.918379)
Supplement: Supplementary file 1 [file DataSheet1.ZIP › Table S1 The primers of eccDNA.docx]

Table S1 The primers of eccDNA

| EccDNA | Forward primer | Reverse primer | Product size |
| --- | --- | --- | --- |
| E1(chr2:16,225,125-16,226,720) | CCTTTGAACTTGCCTGTGCC | AGCCATTTGAACCCCGTTCT | 476 |
| E2(chr10:125,824,765-125,825,185) | ATTGAGGGACGGAAAGGCAG | ATTGAGGGACGGAAAGGCAG | 303 |
| E3(chr23,440,318-23,440,960) | TTCTCCTTCTGTTCCGGCAG | GCAGGAAGATGTGGAGACAG | 500 |
| E4(chr2:88,832,773-88,859,607) | CCTCCTCCTGCCTACTCCTT | CCACTGAGGGCTTGATCAAT | 491 |
