# Supplementary figures and images for: Identification and characterization of extrachromosomal circular DNA in alcohol induced osteonecrosis of femoral head
Source: Front Genet. 2022 Sep 30;13:918379. doi: 10.3389/fgene.2022.918379 (PMC9561878; doi:10.3389/fgene.2022.918379)

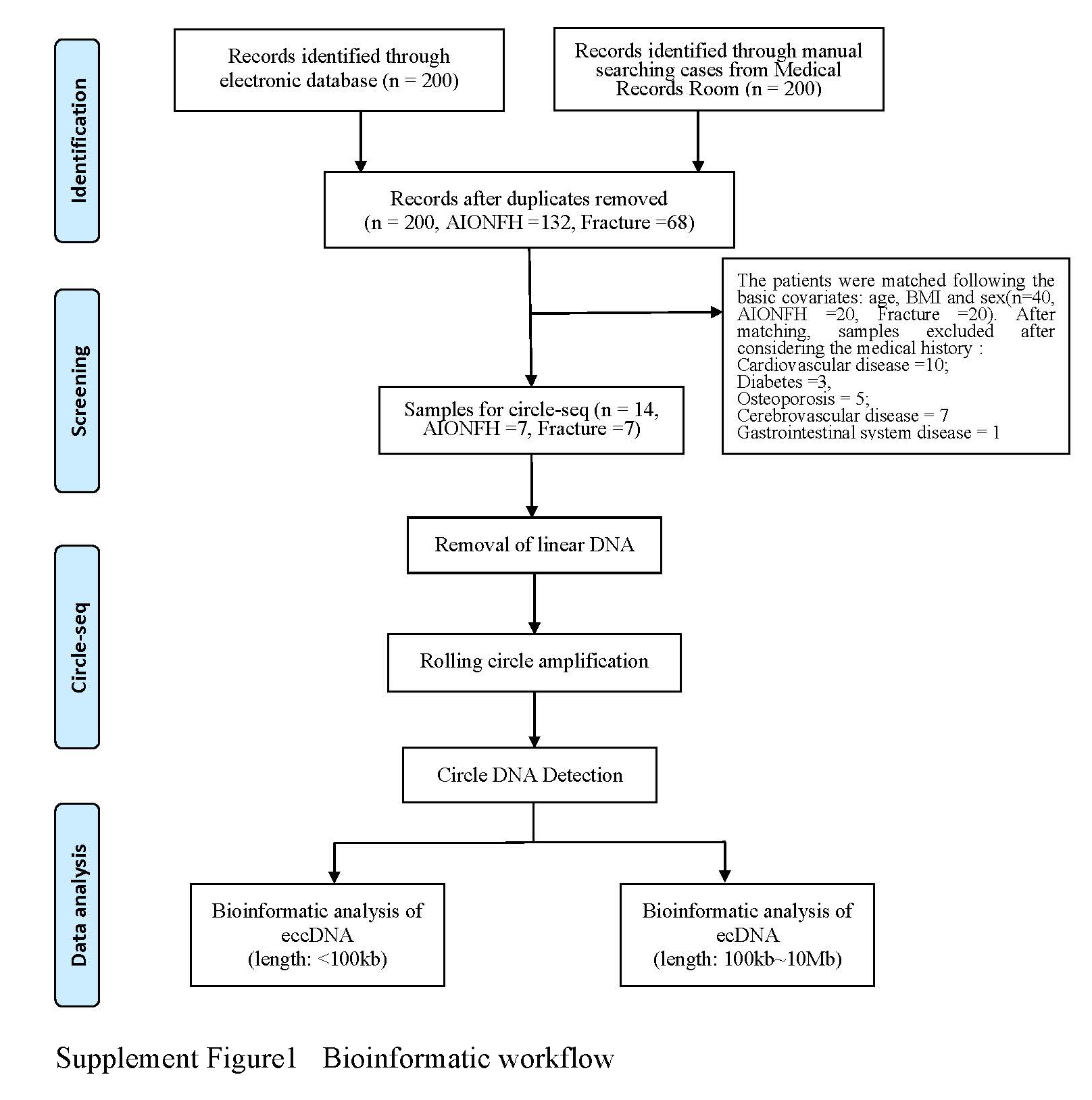

Supplement: Supplementary file 1 [file DataSheet1.ZIP › SUP-F1.jpg]

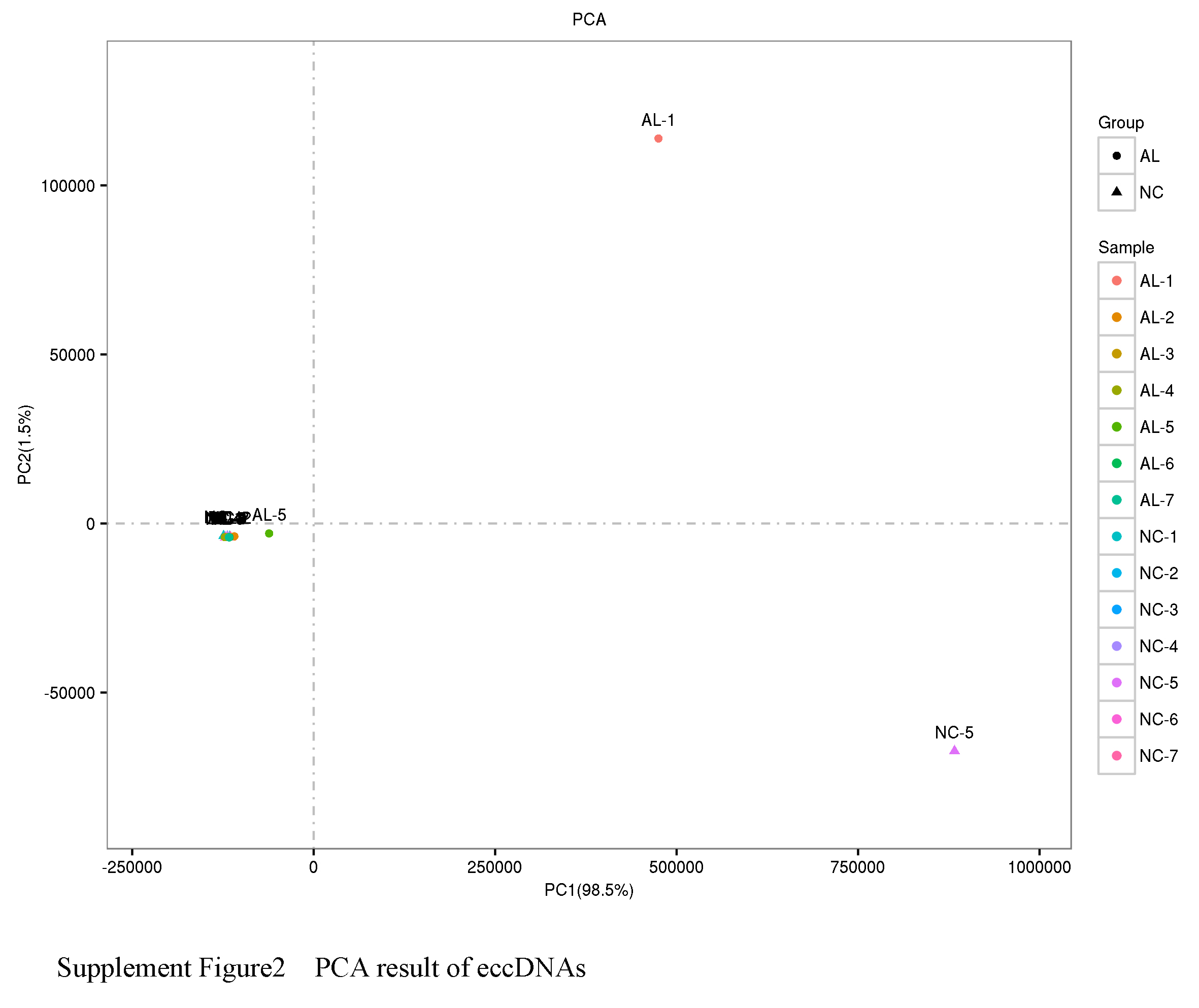

Supplement: Supplementary file 1 [file DataSheet1.ZIP › SUP-F2.jpg]

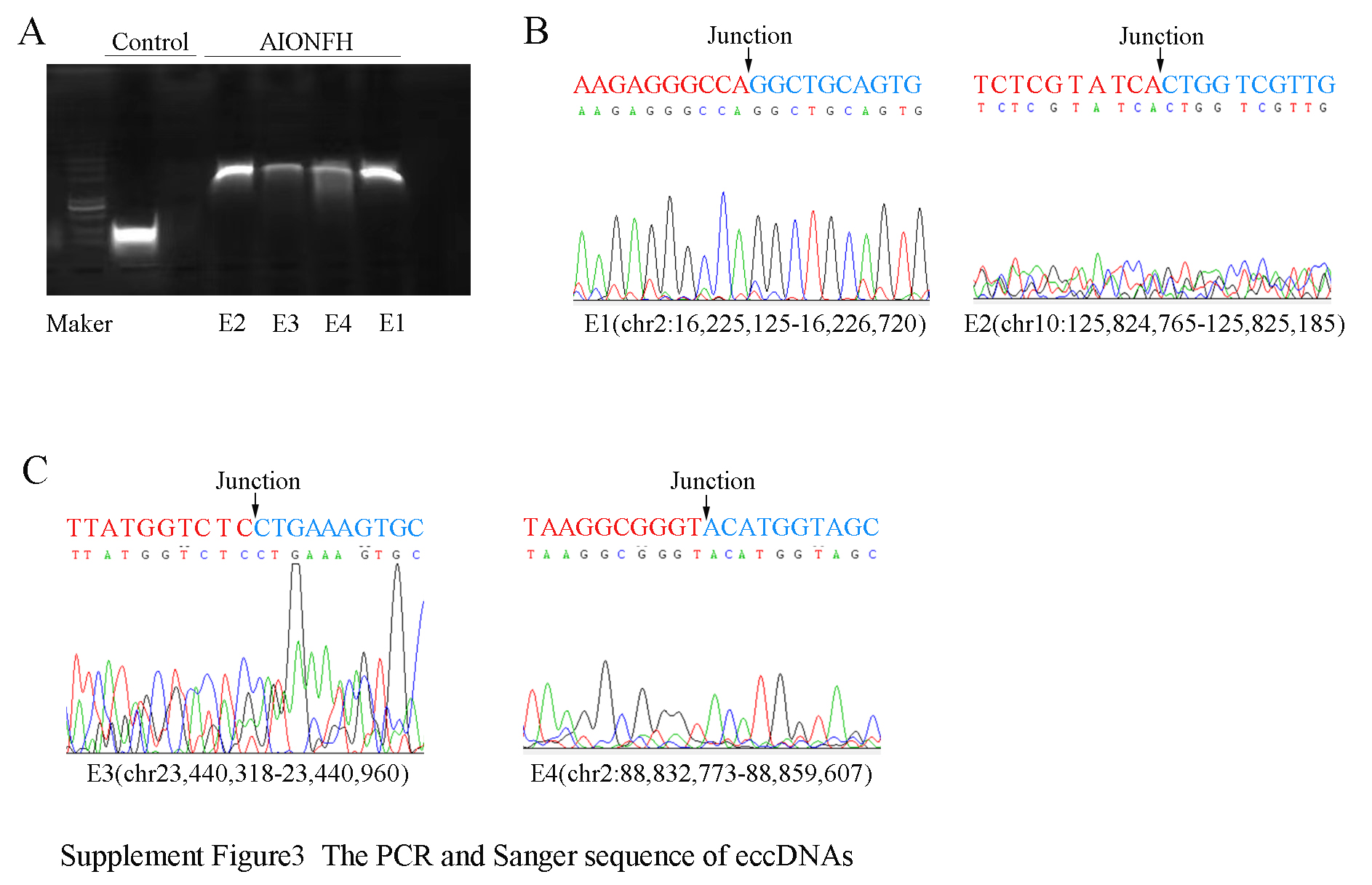

Supplement: Supplementary file 1 [file DataSheet1.ZIP › Sup-F3.jpg]

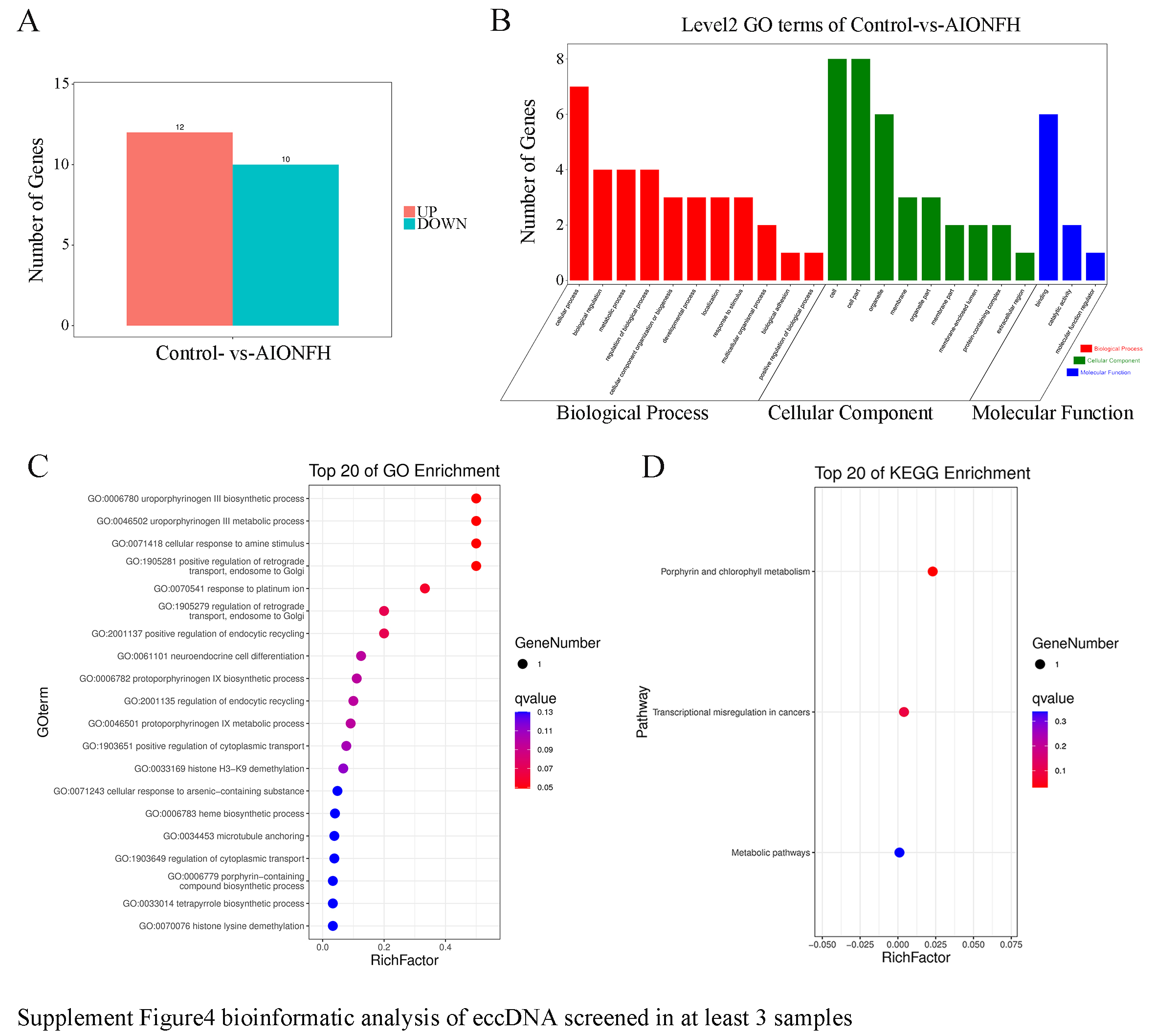

Supplement: Supplementary file 1 [file DataSheet1.ZIP › SUP-F4.jpg]
